# Genetic Control of Water and Nitrate Capture and Their Use Efficiency in Lettuce (*Lactuca sativa* L.)

**DOI:** 10.3389/fpls.2016.00343

**Published:** 2016-03-30

**Authors:** Pauline J. Kerbiriou, Chris A. Maliepaard, Tjeerd Jan Stomph, Martin Koper, Dorothee Froissart, Ilja Roobeek, Edith T. Lammerts Van Bueren, Paul C. Struik

**Affiliations:** ^1^Plant Sciences, Plant Breeding, Wageningen UniversityWageningen, Netherlands; ^2^Plant Sciences, Centre for Crop Systems Analysis, Wageningen UniversityWageningen, Netherlands; ^3^Enza ZadenEnkhuizen, Netherlands; ^4^Enza ZadenSaumur, France

**Keywords:** lettuce, resource acquisition, association mapping, quantitative trait loci, soil sampling, nitrogen use efficiency

## Abstract

Robustness in lettuce, defined as the ability to produce stable yields across a wide range of environments, may be associated with below-ground traits such as water and nitrate capture. In lettuce, research on the role of root traits in resource acquisition has been rather limited. Exploring genetic variation for such traits and shoot performance in lettuce across environments can contribute to breeding for robustness. A population of 142 lettuce cultivars was evaluated during two seasons (spring and summer) in two different locations under organic cropping conditions, and water and nitrate capture below-ground and accumulation in the shoots were assessed at two sampling dates. Resource capture in each soil layer was measured using a volumetric method based on fresh and dry weight difference in the soil for soil moisture, and using an ion-specific electrode for nitrate. We used these results to carry out an association mapping study based on 1170 single nucleotide polymorphism markers. We demonstrated that our indirect, high-throughput phenotyping methodology was reliable and capable of quantifying genetic variation in resource capture. QTLs for below-ground traits were not detected at early sampling. Significant marker-trait associations were detected across trials for below-ground and shoot traits, in number and position varying with trial, highlighting the importance of the growing environment on the expression of the traits measured. The difficulty of identifying general patterns in the expression of the QTLs for below-ground traits across different environments calls for a more in-depth analysis of the physiological mechanisms at root level allowing sustained shoot growth.

## Introduction

Agronomic research has contributed to the design of lettuce cropping systems that maximize yields and optimize quality by supplying abundant water and nutrients, avoiding stress conditions (Gallardo et al., 1996a,b; Broadley et al., 2000; Frantz et al., 2004). In lettuce, drought induced by a shortage in water supply, even temporary, significantly reduces yields, as drought limits shoot growth rate (Biddington and Dearman, 1985; Kerbiriou et al., 2013a). With costs of fossil fuel-based inputs forecasted to increase steadily in the future (Mou, 2011), the environmental and economic sustainability of such intensive systems is becoming more and more questionable, calling for the design of more resilient systems.

Defined as the adaptive capacity to achieve sustainability in a dynamic fashion (Milestad and Darnhofer, 2003), resilience is an important trait of organic farming systems. As organic systems aim at optimizing the production system more than the individual crop, they are considered more resilient than conventional systems which emphasize the productivity of a single crop based on high levels of inputs (Lammerts van Bueren et al., 2011). However, the use of organic manure instead of mineral fertilizer to improve long term soil fertility combined with smaller amounts of irrigation in organic systems, may lead to irregular supply of nutrients and water compromising the certainty of high yields: as soil temperature and moisture conditions affect mineralization of organic matter, crop growth may be more variable in organic systems than in conventional systems which are able to provide the plants with a continuous supply of nutrients available for uptake, though at the expense of potentially large losses to the environment.

Not only improved cultural practices and crop management, but also breeding for robustness—allowing crops to maintain growth despite variable and irregular growing conditions during cropping (Kitano, 2007)—can contribute to the sustainability of more demanding (low input, organic) horticultural systems (Lammerts van Bueren et al., 2002; Wolfe et al., 2008). For instance, new cultivars with more efficient resource uptake and use efficiency may display yield stability under low input or organic farming systems where resource availability is more irregular. Therefore, traits relevant to efficient uptake and use of resources and the possible genetic factors influencing these traits need to be identified.

The traits and the genetics of these traits did not receive much attention in recent breeding programmes of lettuce, a species with nine chromosome pairs. Contemporary approaches have been focusing on breeding for stress tolerance based on head characteristics. For instance, Uwimana et al. (2012) found 17 QTLs associated with vigor in a cultivated (*L. sativa* L.) × wild (*L*. *serriola* L.) lettuce population subjected to drought, salinity and nutrient deficiency. Jenni et al. (2013) found 36 QTLs significantly associated with eight traits linked to heat-stress related physiological disorders in lettuce in recombinant inbred lines derived from an intra-specific cross between two commercial lettuce cultivars.

In lettuce, research on the role of root traits in resource acquisition has been rather limited. As lettuce breeding has been taking place under optimal growth conditions in conventional systems, breeders could afford to select types with a small root system and a high shoot: root ratio, thus increasing harvestable yield (Johnson et al., 2000). Consequently, the root system of modern lettuce varieties is shallow, mainly present in the top 0.2 m of the soil profile where resources are abundant and directly available for uptake in conventional systems (Gallardo et al., 1996b). This morphological feature may affect harvestable yields when these top layers dry out, as no roots are present in the deeper layers of the soil profile where water is available for capture (Jackson, 1995).

One way to improve resource capture and use efficiency and consequently the robustness of new lettuce cultivars may thus be to select for genotypes with a longer, more developed root system able to forage water and nutrients in the lower layers of the soil and compensate for the unavailability of resources in the top layers during a period of drought. With this idea, Johnson et al. (2000) tested whether deeper root foraging and water capture in lower layers of the soil profile were significantly associated with genetic markers in directly sown cultivated (*L. sativa* L.) × wild (*L. serriola* L.) lettuce F_2:3_ families. Thirteen QTLs, each accounting for 28–83% of the phenotypic variation in root traits, were identified, and they showed that the loci for taproot length co-localized with the ability to extract water from deeper soil layers.

However, assessing the genetic diversity of root systems with the objective to breed for improved root system architecture, is very intensive and labor-consuming, especially under field conditions where roots have to be sampled, washed, manually cleaned to remove organic litter and scanned. Instead, it might be easier to take soil samples to measure resource capture, and by a modeling approach, predict root characteristics—based on the assumption that root characteristics and resource capture are strongly correlated within relevant ranges, as shown by King et al. (2003) in barley and surmising that nitrogen accumulation in the heads is correlated with resources removed from the soil.

Kerbiriou et al. (2014) showed that in lettuce the relationship between root mass and nitrate capture does not follow the relationship found by King et al. in barley (King et al., 2003), where the non-captured resource logarithmically declines with an increase in the amount of roots or with the root length density. Although nitrate capture in lettuce is generally fairly correlated to root mass or root length density when field conditions are conducive to growth (Kerbiriou et al., 2013a), in lettuce localized root growth is related to specific, localized resource availability as demonstrated by Kerbiriou et al. (2013b) in a pot trial. In case localized nitrate shortage was applied, root growth was more abundant in N rich soil layers—as previously noted by Hodge (2004) in grass species under various conditions—whereas when localized drought was applied, root growth occurred in the dry compartment (as opposed to the moist compartment). These findings highlighted that the relationship between root growth and resource capture in lettuce is complicated, and requires a novel modeling approach before resource capture can be related to root traits—as discussed by Kerbiriou et al. (2014).

This study by Kerbiriou et al. (2014) also revealed that large genetic variation can be found in the temporal and spatial dynamics of resource capture below-ground and use of these resources above-ground. The patterns of nitrate and water capture in 0.1 m soil layers over a 0.4 m soil profile in a population of 148 lettuce cultivars grown in four environments proved to be highly diverse and complex, supporting the idea that it would be possible but difficult to breed for traits related to below-ground performance.

While the mechanisms involved in resource capture and use were analyzed in Kerbiriou et al. (2014), the current paper addresses the genetic control of such traits, in other words explores the association between the phenotypic traits involved in resource capture and use efficiency, and genotyping information provided by Single Nucleotide Polymorphism (SNP) markers. With this objective, a population of 148 lettuce cultivars was planted during two seasons (spring and summer) in two different locations under organic cropping conditions, and nitrate and water capture below-ground and in the shoots were assessed during growth and when the plants reached a harvestable size. Simultaneously, 1170 SNP markers were scored for each cultivar using the KASP™ technology (LGC Genomics, Hents, UK). The statistical significance of the association between the measured traits and the markers was tested with the aim to find QTLs associated with nitrate and water capture and use efficiency, and to understand their interaction with the growing environment. A complete set of reliable data was obtained for 142 cultivars.

## Materials and methods

### Cultivar choice

Two-hundred-fifty lettuce accessions, commercially available in the period between 1960 and 2008 were grown under field conditions in 2008 and were evaluated for a broad range of crop growth parameters. Out of these 250 accessions from various seed companies, 148 butterhead types suitable for field cultivation under either spring or summer conditions, or both, were selected for this study. Criteria for selection included diversity in head characteristics (large vs. compact heads, color, leaf shape, leaf texture, etc.), commercial origin (seed company), and country and date of release. Criteria for selection did not include traits related to root characteristics, as they were not known for the entire set of cultivars, but we surmised that cultivars released before 1970 had larger root systems and lower harvest indices than more recent cultivars. In the selected population, 27 cultivars were released before 1970, 24 cultivars were released between 1970 and 1990, and 95 cultivars were released after 1990; the time of release of two cultivars was unknown. Eight cultivars were known to be grown by amateur gardeners, and two cultivars came from breeding programmes targeting specifically organic farming systems.

### Transplants raising and transplanting

Seeds used originated from randomly selected plants from the screening trial in 2008. Prior to transplanting, seeds were sown in 4 × 4 × 4 cm organic peat blocks (Jongerius, Houten, the Netherlands) after breaking seed dormancy by exposure to 4°C for 24 h. Transplants were raised in a greenhouse with a day temperature of 20°C and a night temperature of 15°C.

Transplanting was done when the transplants had 5–7 leaves and few roots started to emerge out of the peat block. In the field, plant arrangement was 0.3 × 0.3 m.

### Experimental design

**Figure 1:**
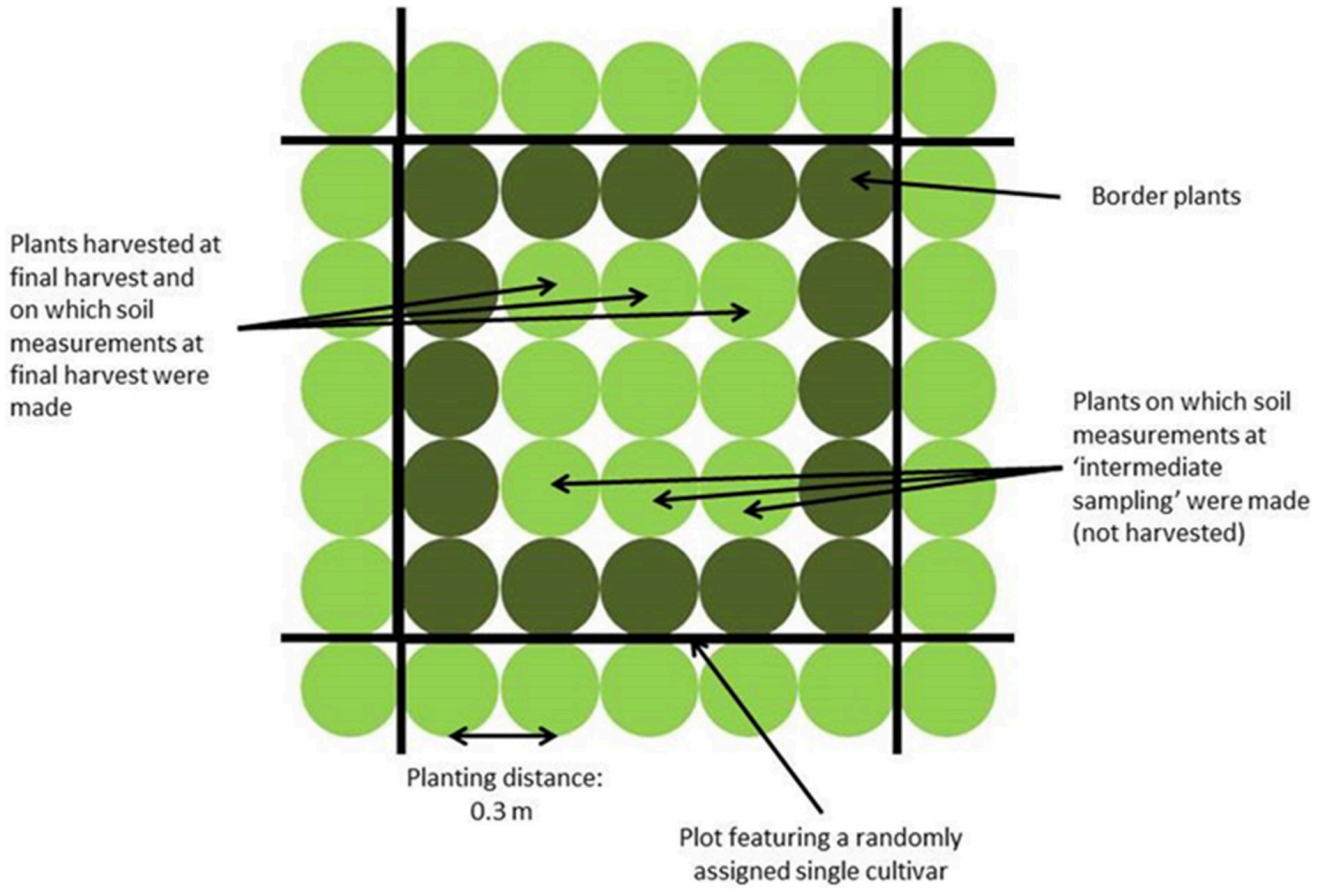
**Sampling scheme for a plot featuring a single cultivar**.

Four field trials were performed: two in Wageningen (51.97° N, 5.67° E, The Netherlands), in spring and summer 2010, and two in Voorst (52.23° N, 6.08° E, The Netherlands), in spring and summer 2011. Each trial included two repetitions. The experimental set up was a randomized complete block design, each block consisting of 150 plots. Two plots per block were left empty for measurements in bare soil. Not bare plots were planted to 25 plants (5 × 5 plants) of the same cultivar (cf. Figure 1). Plants at the beginning and end of each row were considered guard plants; therefore measurements were done on the nine (3 × 3 inner) plants of the net plot.

### Field management

Field sites were chosen according to their soil quality (uniform soil profile up to 0.5 m depth and a physical structure allowing undisturbed root growth) and previous crop management. All sites had been cropped uniformly in the previous 5 years on a larger surface than the area covered by the trials, in order to avoid influence of previous crops or field management on soil characteristics. In both locations the soil was sandy, poor with a low content in organic matter (8–10%), and low water retention capability. All trial fields were certified organic and managed according to organic standards during the experiments.

For even distribution of nutrients, fertilization was provided by applying 100 kg/ha nitrogen, from seaweed pellets (9% N, 3% P, 3% K + 3% MgO, EcoFertiel™, EcoStyle, Appelscha, The Netherlands) on the day before transplanting, instead of using compost or manure. Weeding was done manually every week. Irrigation was not applied.

### Field conditions

**Figure 2:**
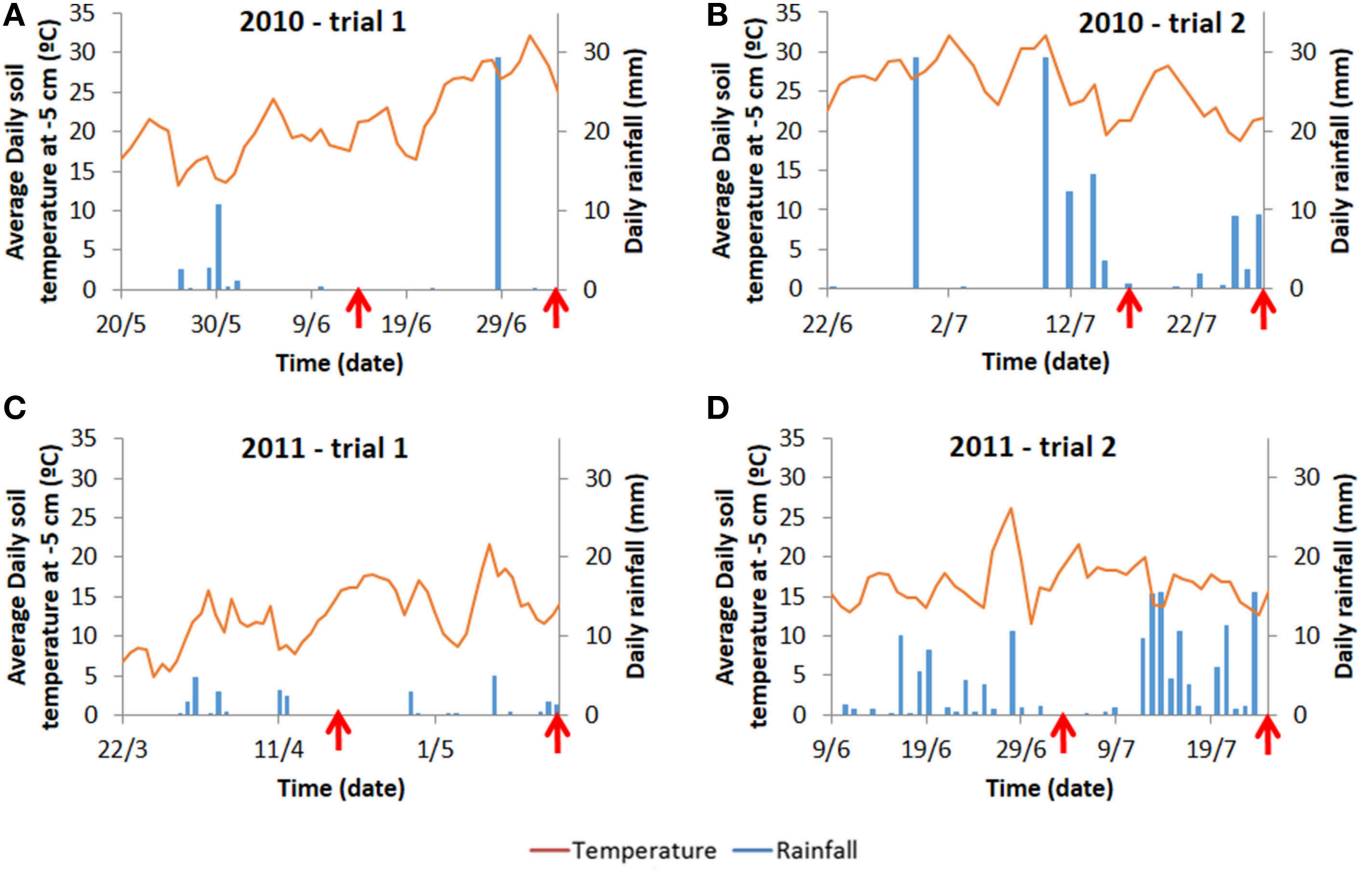
**Average daily temperature recorded at 5 cm below ground and average daily rainfall for the Wageningen trials (A,B) and for the Voorst trials (C,D)**. Daily temperatures at −5 cm are provided as they are more prone to fluctuations than temperatures at deeper layers reflecting more strongly the variable conditions above ground. Moreover, −5 cm is also the depth at which initial root growth starts on transplants. Arrows indicate the time at which intermediate sampling and final harvest occurred (cf. Materials and Methods).

For each trial, weather data (air temperature, radiation, rainfall) were recorded daily (Voorst) or hourly (Wageningen) at the nearest weather station (for the Wageningen trials, data were collected from http://www.met.wau.nl/ and for the Voorst trials, data were collected from the on-farm weather station). Soil temperatures were measured in 4 horizons (0.0–0.1, 0.1–0.2, 0.2–0.3, 0.3–0.4 m) using a data logger. Cumulative degree-days (based on air temperatures, calculations see below), as well as cumulative rainfall at each sampling date for each trial are shown in Table 1. Details of daily temperature fluctuations and daily rainfall events are shown in Figure 2.

**Table 1:** **Transplanting, intermediate sampling and final harvest dates, and weather conditions during the four experiments**.

**Year**	**2010**				**2011**			
**Location**	**Wageningen**				**Voorst**			
	**Trial 1**		**Trial 2**		**Trial 1**		**Trial 2**	
**Planting date**	**20-05-2010**		**22-06-2010**		**22-03-2011**		**09-06-2011**	
	**Intermediate sampling**	**Final harvest**	**Intermediate sampling**	**Final harvest**	**Intermediate sampling**	**Final harvest**	**Intermediate sampling**	**Final harvest**
Sampling date	14-06-2010	05-07-2010	19-07-2010	28-07-2010	19-04-2011	17-05-2011	05-07-2011	25-07-2011
Cumulative rainfall (mm)	18	48	90	104	16	27	49	145
CDD^*^ (°Cd)	357	793	607	782	174	481	329	590

* Cumulative degree-days (using 4°C as base temperature).

### Phenotyping

#### Calculation of thermal time

Cumulative degree days at each sampling date were calculated as the sum, between the date of transplanting and the sampling date, of the degrees above 4°C (base temperature for lettuce; Kristensen et al., 1987), based on average daily temperature:

$${\text{CDD}}_{\text{sampling x}}=\sum_{day\;0}^{sampling\;date\;x}\left[\frac{\left(T_{max}\;+\;T_{min}\right)}{2}-T_{base}\right]$$

where T_max_ and T_min_ correspond respectively to the maximum and to the minimum temperatures recorded on a certain day and with T_min_ > T_base_.

#### Soil measurements

Soil samples were taken every 0.1 m over a depth of 0.4 m outside of the peat block, using a 0.06 m diameter and 0.40 m long auger, during growth (“intermediate sampling”) and at final harvest (“final sampling”). For three plants per plot, soil samples taken in each soil layer were pooled to account for plant-to-plant variation.

Volumetric soil moisture content (% v:v) was recorded after drying at 40°C for 48 h.

Nitrate content (soil [NO_3_], assessed in ppm) in each 0.1 m soil layer was measured using an Ion Selective Electrode (ThermoFisher™, Waltham, MA, USA) using the method described previously by Sibley et al. (2009) and also used in Kerbiriou et al. (2013a).

#### Shoot measurements

Shoot measurements were done only at final harvest. Fresh weight and dry weights (g per plant) were assessed based on three plants per plot at final harvest, which took place 5–9 weeks after transplanting depending on the trial (for sampling method, see Figure 1). The averages over six plants per cultivar per trial (three plants per replicate, two replicates per trial) were used in the association mapping study. Nitrogen concentration (g N per kg dry matter) in the head was measured using the Kjeldahl method, based on the ground material of three plants per cultivar and per replicate within a trial. Physiological Nitrogen Use Efficiency (NUE, g DM per g N in head) was calculated based on the head [N] as NUE = 1/(head [N]). The average value over the two replicates within a trial was used for the association mapping study.

### Analysis of variance of phenotypic data

Supplementary Material Table S1 contains all data on shoot and below-ground characteristics. Soil and shoot phenotypic data were statistically analyzed as described in Kerbiriou et al. (2014).

### Heritability

The genotypic and residual variance components were estimated using the Residual Maximum Likelihood Estimations (REML) analysis of Genstat 15th Edition (Hempstead, UK) with the following mixed model: response = general mean + genotype + block + error. Heritability (h^2^) estimates were then calculated based on the variance components as follows: h^2^ = $\sigma_{\text{g}}^{2}$/($\sigma_{\text{g}}^{2}+\sigma_{\text{e}}^{2}$) where $\sigma_{\text{g}}^{2}$ was the estimate of the genotypic variance and $\sigma_{\text{e}}^{2}$ was the residual variance.

### Genotyping

Lettuce DNA was isolated from leaf material taken when they had reached the 5th leaf stage; these plants were specifically grown in a greenhouse for the purpose of genotyping. The plants were grown from seeds originating from the same seed lot as was used for the phenotyping experiments.

Single Nucleotide Polymorphisms (SNPs) were mined from various transcriptome sequencing projects done on the leaves of two lettuce lines (proprietary markers by Enza Zaden). SNPs were identified in lettuce Expressed Sequence Tags (EST) and only the 1348 SNPs with high probability scores were conferred into KASP™ assays (LGC Genomics, Hents, UK). Six cultivars from the 148 cultivars tested in the field were discarded in the association mapping studies because of large amounts of missing values (more than 10%) for these cultivars, therefore only 142 cultivars in total were kept in the analyses. Markers of poor quality or rare alleles (less than 10% occurrence) were also removed from the analysis and at the end 1170 markers were used. SNP markers were run with the DNA of the lettuce population on a Fluidigm chip on a Biomark HD system (Fluidigm™, San Francisco, USA). The percentage of heterozygous scores over all SNPs and all accessions was 0.87%, with one accession having 6.6% heterozygosity over all SNPs and the remaining accessions all having less than 1.5% heterozygosity. The SNPs were mapped to nine linkage groups (corresponding to the linkage group numbering in the consensus integrated map of Truco et al. (2007, 2013), plus a residual set of markers of which the map positions were unknown. The average distance between markers was 0.4 cM. A summary of genotypic information is given in Table 2 and the genotype scores and linkage map are given in Supplementary Material Table S2.

**Table 2:** **Summary of marker information**.

**Chromosome**	**Length (cM)**	**Number of markers**	**Median distance between markers**	**95% percentile of distance**
1	132	171	0.2	4.7
2	124	89	0.5	6.1
3	92	65	0.7	6.4
4	162	209	0.3	3.3
5	156	172	0.3	4.4
6	98	36	0.5	15.1
7	112	118	0.4	4.0
8	169	166	0.3	4.7
9	97	67	0.2	8.3
Unmapped	76	77		
Genome	1217	1170	0.4	4.6

### Association mapping procedure for QTL detection

#### Principal components analysis (eigenanalysis)

Population structure was investigated following the approach by Price et al. (2006) and Patterson et al. (2006) using the QEIGENANALYSIS procedure in Genstat 15th Edition (Hempstead, UK) and the 1170 SNP markers set. Seventeen significant eigenvectors were obtained and used as covariates to account for population structure in the marker-trait association models.

#### Linkage disequilibrium (LD) decay investigation

**Figure 3:**
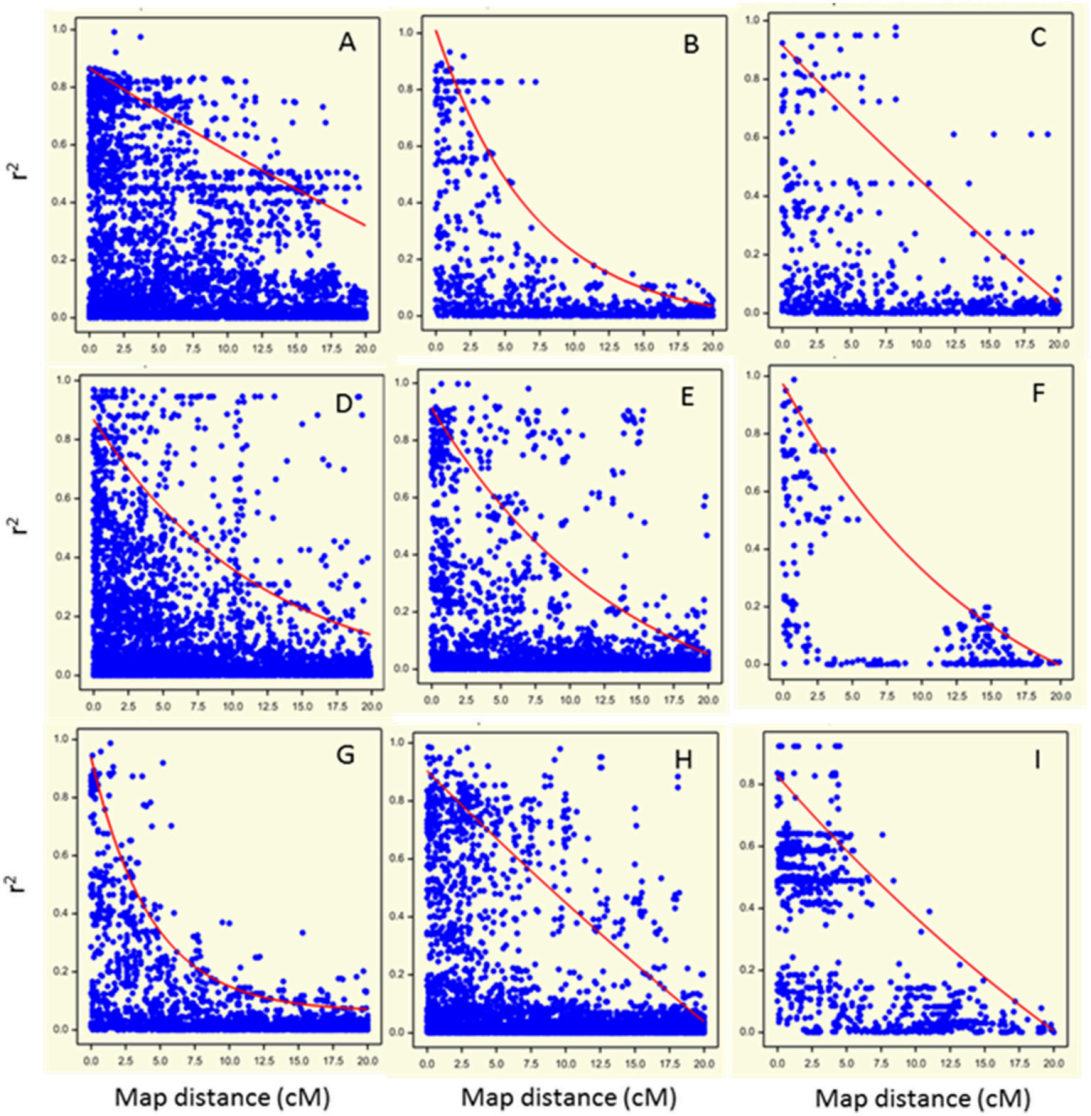
**Visualization of Linkage Disequilibrium (LD) decay as the squared coefficient of the relation between two markers (*r*^2^) plotted against the genetic distance between two markers in cM (dots) for each chromosome (A: chromosome 1; B: chromosome 2; C: chromosome 3; D: chromosome 4; E: chromosome 5; F: chromosome 6; G: chromosome 7; H: chromosome 8; I: chromosome 9)**. The trend line illustrates the LD decay based on the non-linear regression of the *r*^2^ on genetic distance.

Marker-marker associations (LD decay) were investigated on the set of 1170 SNP markers correcting for relatedness using the significant eigenvectors as covariates in the QLDDECAY procedure in Genstat 15th Edition (Hempstead, UK). For each chromosome, pairwise LD between markers was calculated using the square of the corrected correlation coefficient, *r*^2^ (Pritchard and Przeworski, 2001). These squared corrected correlation coefficients were plotted against the genetic distance between markers (in cM) to evaluate LD decay, separately for each chromosome (Figure 3).

#### Association mapping analysis

All the mean shoot and soil measurements obtained for each cultivar in each environment were used as phenotypic data to be related to the genotypic data. Association mapping studies were carried out for each trait at each sampling date within each environment using the QASSOCIATION procedure in Genstat 15th Edition (Hempstead, UK). Population structure was corrected for using the eigenvectors as random covariates in a linear mixed model with QTL as fixed effect at the marker position. The Wald-test was used to test significance; Wald *p*-values were –log_10_ transformed. To account for multiple testing, a number of effective tests (# tests) was calculated as the ratio of the total genome size to the average LD over the nine chromosomes, and used to calculate the threshold of significance to claim a significant QTL as: threshold = −log_10_(0.05/# tests). Because a threshold of 3.5 was more stringent than a 5% false discovery rate, this value was used to identify significant marker-trait associations throughout the analyses.

The threshold for the minor allele frequency (MAF) was set to 7% (at least 10 accessions should have the minor allele) for testing marker-trait associations.

## Results

### Phenotyping results

**Figure 4:**
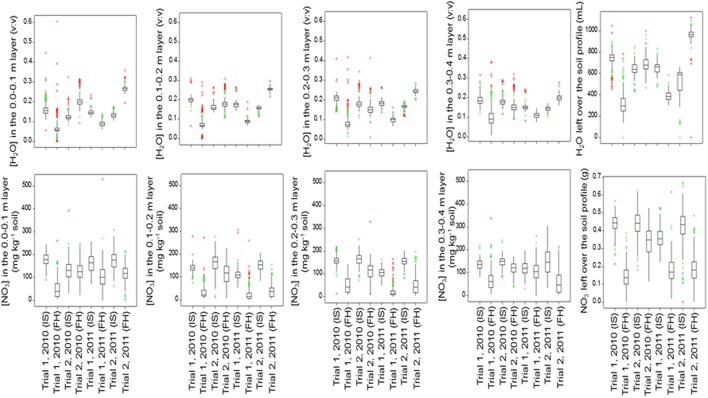
**Boxplots of the below-ground traits for the population of 142 lettuce cultivars in each trial and at each sampling date (IS, Intermediate Sampling; FH, Final Harvest)**.

**Figure 5:**
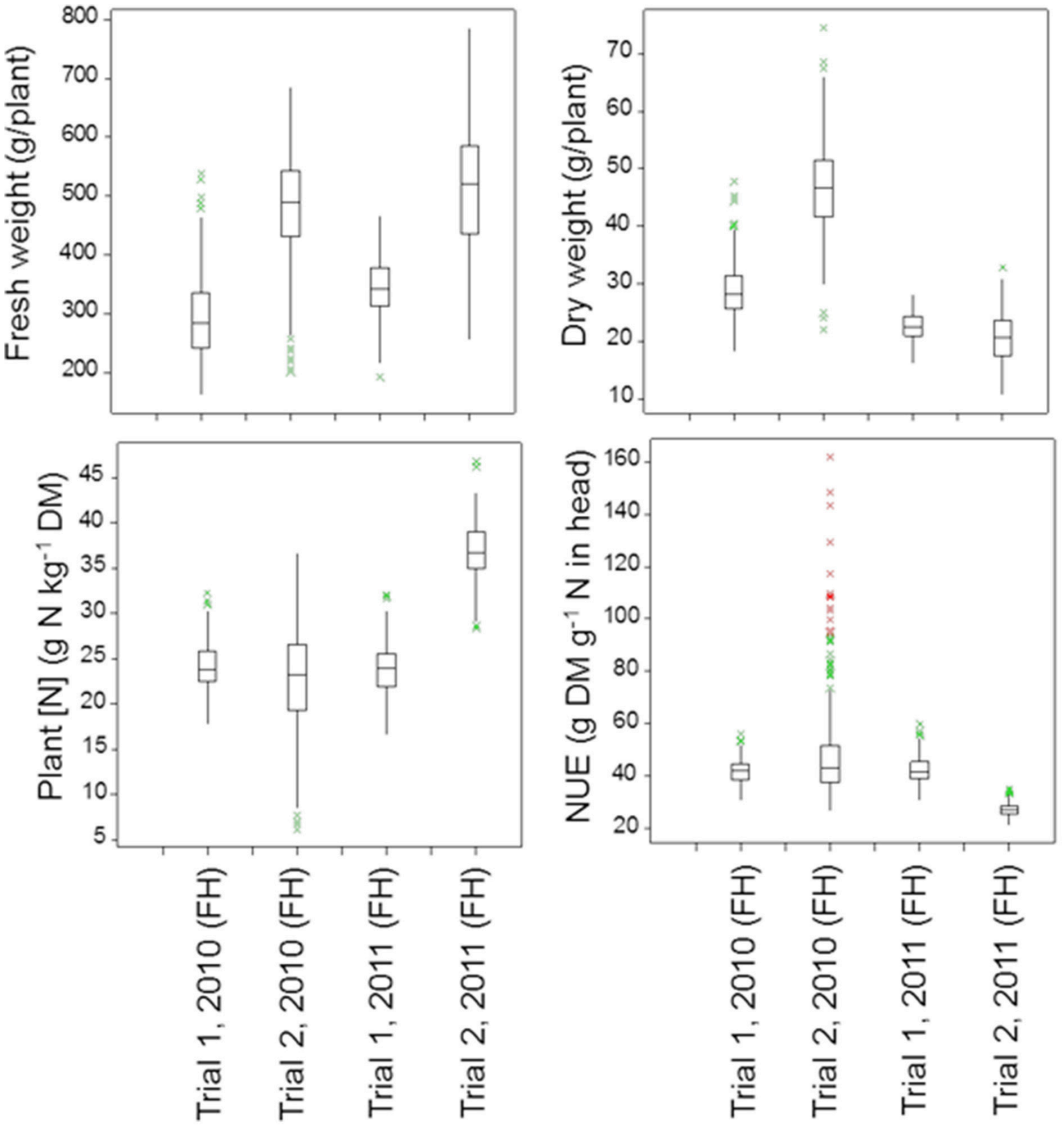
**Boxplots of the shoot traits for the population of 142 lettuce cultivars in each trial (FH, Final Harvest)**. The *P*-values for these shoot traits in the four trials are as follows:

Figure 4 (below-ground traits at both sampling dates) and Figure 5 (shoot traits at final harvest) summarize the mean values and genetic variation in the population of the 142 cultivars used in this study. Which variables showed significant genetic variation is indicated in Table 3 (bold numbers for heritability).

**Table 3:** **Heritability (%) of soil and plant measurements at intermediate (Inter.) and final (Final) sampling for the four trials across the population of lettuce**.

	**2010**				**2011**			
	**Trial 1**		**Trial 2**		**Trial 1**		**Trial 2**	
**Sampling**	**Inter**.	**Final**	**Inter**.	**Final**	**Inter**.	**Final**	**Inter**.	**Final**
**SOIL [H_2_O] LEFT IN LAYER (v:v)**								
0.0–0.1 m	9	0	9	**21**	0	**14**	0	**15**
0.1–0.2 m	0	1	0	0	0	**25**	**7**	0
0.2–0.3 m	0	7	6	9	0	**38**	**4**	0
0.3–0.4 m	0	2	3	0	2	**16**	0	0
**WATER LEFT OVER THE 0.4 m SOIL PROFILE (mL)**								
	0	9	**18**	13	0	**30**	2	**15**
**Soil [NO_3_] left in layer (mg kg^−1^ soil)**								
0.0–0.1 m	0	0	5	**25**	0	3	0	0
0.1–0.2 m	0	0	1	**17**	0	0	0	**15**
0.2–0.3 m	0	0	**14**	**16**	**13**	0	**21**	**16**
0.3–0.4 m	0	2	9	7	0	0	**9**	**17**
**NO_3_ LEFT OVER THE 0.4 M SOIL PROFILE (g)**								
	0	0	5	**23**	1	4	0	9
Plant fresh weight (g)		8		**48**		**38**		**55**
Plant dry weight (g)		**14**		**27**		**52**		**48**
Plant [N] (g N kg^−1^ DM)		**43**		5		**46**		**17**
Plant NUE (g DM g^−1^ N in head)		**39**		2		**43**		**18**

For values in bold the genetic variation as illustrated in the boxplots of Figures 4, 5 was statistically significant at p ≤ 0.05.

Moisture or nitrate measurements in the soil at intermediate or final sampling did not show significant variation caused by cultivar differences for Trial 1, 2010, with relatively mild temperatures and dry weather. Significant genetic variation was found in moisture content in each soil layer and over the whole soil profile at final sampling in Trial 1, 2011, with relatively low temperatures and dry weather; nitrate left in the soil did not show much genetic variation in Trial 1, 2011. Highest levels of nitrate left in each soil layer and over the whole soil profile at final harvest were recorded for Trial 2, 2010, under optimal growing conditions, with in most cases significant genetic variation. Also in Trial 2, 2011, an experiment under conducive growing conditions, several soil variables showed significant genetic variation (Figure 4; Table 3).

Under optimal growing conditions (Trial 2, 2010) the highest dry matter production and highest Nitrogen Use Efficiency (NUE) at final harvest were achieved (Figure 5). Significant genetic variation was found in fresh and dry yields. No significant genetic variation was found in plant nitrogen or NUE in this trial. Under dry conditions (Trial 1, 2010, and Trial 1, 2011), genetic variation was found in all shoot measurements at final harvest, except for fresh yield in Trial 1, 2010. Trial 2, 2011, had the highest values for plant nitrogen, with relatively small, but significant genetic variation (Figure 5; Table 3).

### Heritability of the traits

Per trial, the heritability estimates were low for the soil moisture content measurements at each layer and over the whole soil profile, except for the measurements made at final harvest in Trial 1, 2011 (moderately dry conditions), where estimates ranged from 14 to 38% (Table 3).

The heritability in the [NO_3_] traits was the largest at final harvest in Trial 2, 2010 (optimal growing conditions), and Trial 2, 2011 (wet conditions), with values ranging from 7 to 17% in the layers 0.1–0.4 m of the soil (Table 3).

Shoot traits (plant fresh and dry weights, plant [N] and plant NUE) were generally the traits for which the heritability was the largest, with values up to 55% (Table 3). The highest heritabilities for shoot traits were obtained in the trials in Voorst 2011, with values ranging from 17 to 55%, compared to the trials carried out in Wageningen in 2010 where values ranged from 2 to 48%.

### LD decay analysis

Pairwise LD decreased rapidly with genetic distance on all chromosomes except chromosome 1 (Figure 3). For chromosomes 1, 3, 4, 5, and 8 (Figures 3A,C,D,E,H, respectively), regions of high LD were mixed with regions of low LD. Basal LD, defined as the critical value of *r*^2^ beyond which LD was assumed to be due to genetic linkage, was estimated to be 0.2 over the whole genome. For each chromosome, intra-chromosomal LD was calculated as the intersection of the LD trend line with the basal *r*^2^ (Figure 3). Intra-chromosomal LD was found to decay between 8 and 17 cM for individual chromosomes (except for chromosome 1 where it was at about 35 cM) and average LD decay over the whole genome was estimated at 15 cM. Note that the pattern for chromosome 6 is deviating due to its low number of (widely spaced) markers.

### Marker-trait associations

Many significant QTLs were found in the association mapping study, especially for the traits measured at final harvest. Most of the QTLs found were located on chromosomes 4, 5, 7, and 9, while only very few significant associations were found on chromosomes 1, 2, 3, 6, and 8 (Tables 4–**6**).

**Table 4:** **Significant marker-trait associations (−Log_10_(P) >3.5) for overall NO_3_ left over the soil profile (g) at final sampling, and their position (cM) on the lettuce chromosome (Chr.) identified in each environment (Year × Trial combination) with –Log_10_(P) score, allele frequency (Allele fq.), allele effects and absolute value of the standard error (SE)**.

**Year**	**Trial**	**Chr**.	Marker^*^	**cM**	**−Log_10_(P)**	**Allele fq. (%)**	**Allele effect**	**SE**
2011	1	4	LSM00408	79.3	3.74	88.0	0.024	0.006
2011	1	4	LSM00032	80.1	3.72	86.4	0.024	0.006
2011	1	4	LSM01321	83.2	4.52	89.4	0.028	0.007
2011	2	4	LSM00408	79.3	4.19	88.0	0.034	0.008
2011	2	4	LSM00496	80.5	4.37	87.1	0.031	0.008
2010	2	5	LSM00319	92.3	4.02	92.9	0.007	0.000
2010	2	7	LSM00610	43.6	5.52	92.2	0.055	0.012

* SNPs with their flanking sequences in Supplementary Material Table S3.

#### Below-ground traits

NO_3_ left both over the full soil profile and in each soil layer showed the highest counts of significant marker-trait associations across environments (cf. Tables 4, 5); there significant associations were consistent across trials and over the different layers of the soil profile. Contrastingly, significant marker-trait associations for water left over the soil profile were found only in Trial 2, 2011 (on chromosome 4 at 88.6 cM; on chromosome 5 at 92.3 cM; on chromosome 9 at 53.8 and 58.0 cM) and in Trial 2, 2010 (on chromosome 1 at 68.3 cM and on chromosome 7 on 43.6 cM).

**Table 5:** **Significant marker-trait associations (−Log_10_(P) > 3.5) for [NO3] left in a layer (ppm) at final sampling and their position (cM) on the lettuce chromosome (Chr.) identified in each environment (Year × Trial combination) with –Log_10_(P) score, allele frequency (Allele fq. in %), allele effects (ppm) and absolute value of the standard error (SE)**.

**Year**	**Trial**	**Layer**	**Chr**.	Marker^*^	**cM**	**−Log_10_(P)**	**Allele fq. (%)**	**Allele effect**	**SE**
2010	2	0.3–0.4 m	4	LSM00409	42.4	3.52	84.6	7.1	2.0
2010	2	0.2–0.3 m	4	LSM00408	79.3	3.75	88.0	13.4	3.6
2010	2	0.2–0.3 m	4	LSM00496	80.5	4.63	87.1	14.6	3.5
2010	2	0.2–0.3 m	4	LSM00344	84.8	3.91	86.4	13.1	3.4
2011	2	0.2–0.3 m	4	LSM00408	79.3	8.42	88.0	24.3	4.1
2011	2	0.3–0.4 m	4	LSM00496	80.5	10.8	87.1	29.0	4.3
2011	2	0.2–0.3 m	4	LSM01560	81.3	5.27	86.5	19.3	4.3
2011	2	0.2–0.3 m	4	LSM00434	82.3	4.62	87.2	18.7	4.4
2011	2	0.3–0.4 m	4	LSM00344	84.8	6.34	86.4	25.0	4.9
2010	2	0.1–0.2 m	5	LSM00319	92.3	6.31	92.9	30.5	6.1
2010	2	0.0–0.1 m	7	LSM00610	43.6	12.8	92.2	31.1	4.2
2010	2	0.3–0.4 m	7	LSM01558	94.2	3.88	69.3	5.7	1.5
2010	2	0.3–0.4 m	7	LSM01772	97.2	3.89	67.9	5.7	1.5
2011	2	0.1–0.2 m	7	LSM00610	43.6	4.25	92.2	15.8	3.9
2011	2	0.2–0.3 m	9	LSM00232	53.7	7.62	90.8	20.7	3.7
2011	2	0.2–0.3 m	9	LSM00443	54.3	7.15	91.3	20.8	3.9
2011	2	0.2–0.3 m	9	LSM00605	55.0	5.63	92.1	19.2	4.1
2011	2	0.2–0.3 m	9	LSM01377	57.7	6.07	91.4	19.0	3.9
2011	2	0.2–0.3 m	9	LSM01604	58.0	5.68	92.3	19.2	4.0
2011	2	0.2–0.3 m	9	LSM01220	58.7	6.96	89.0	18.1	3.4

* SNPs with their flanking sequences in Supplementary Material Table S3.

As shown in Table 4, significant marker-trait associations for NO_3_ left over the full soil profile were found on chromosomes 4, 5, and 7 and mostly at final harvest. The frequencies of the major allele for these markers were high over the population, with frequencies ranging from 92.2 to 86.4%. The effects of these QTLs were intermediate, with approx. 15% difference in overall NO_3_ content over the whole soil profile between the two parts of the population bearing the different alleles.

The same was true for the marker-trait associations tested for the [NO_3_] in the different soil layers (cf. Table 5). The frequency of the major allele for these markers was also high among the population with values above 65%. The effect of the QTL located in the region around 80 cM on chromosome 7 was intermediate to high, with in Trial 2, 2010, 11% difference and about 40% difference in Trial 2, 2011, between the cultivars bearing one allele and the cultivars bearing the other allele. The effect of the QTL located between 50 and 60 cM on chromosome 9 was also moderate with about 30% in [NO_3_] in the considered layers between the part of the population bearing one allele compared to the part of the population bearing the other allele. Only the significant QTLs detected at final harvest are displayed in Table 5.

Significant marker-trait associations were identified for moisture content in specific layers only in Trial 2, 2011, at final harvest for layer 0.1–0.2 m on chromosome 7 (69.8 cM) and chromosome 9 (52.0 cM), for layer 0.2–0.3 m on chromosome 9 (53.7 and 57.7 cM) and for layer 0.3–0.4 m on chromosome 7 (97.2 cM).

**Figure 6:**
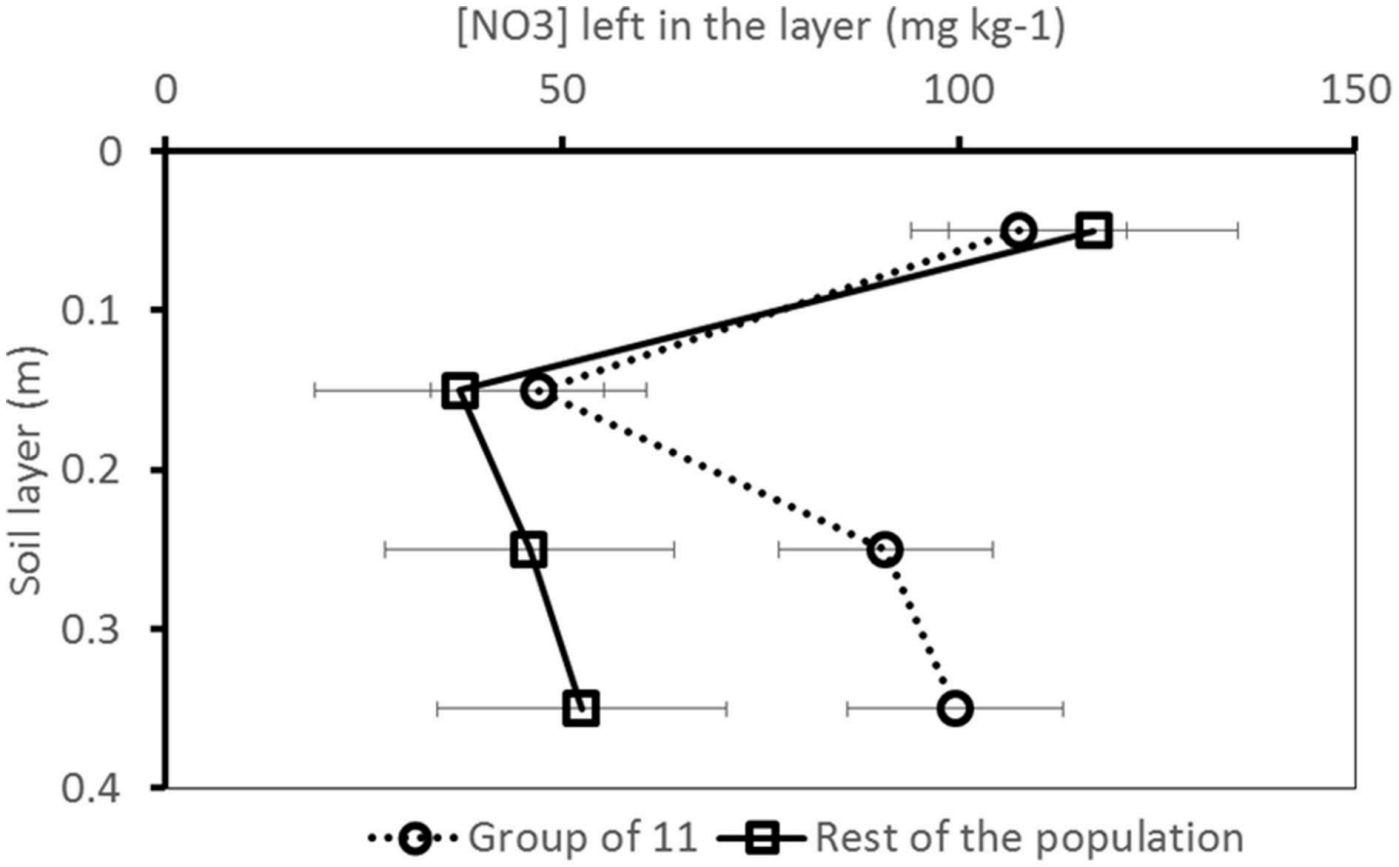
**[NO_3_] pattern over the soil profile for the group of 11 cultivars bearing a different allele for the significant markers identified on chromosome 9 compared to the rest of the population (Trial 2, 2011, final harvest)**. Whiskers indicate standard deviation.

Because of the large number of QTLs detected on chromosome 9, we had a closer look on this region, and we identified a group of 11 cultivars bearing a different allele than the rest of the population for the detected markers and traits. This cluster was composed of 4 cultivars released before 1970, 3 cultivars released between 1970 and 1990 and 5 cultivars released after 1990. They came from a gene bank (5), from a single seed company (3), or from diverse seed companies (3). The ANOVA based on this grouping showed that this cluster left significantly more H_2_O and NO_3_ (*p*-value ≤ 0.05) in the deeper soil layers than the rest of the group (Figure 6). The cultivars in this group also had significantly lower fresh and dry yields.

#### Shoot traits

Several significant QTLs were detected for the shoot traits, mainly for the shoot fresh and dry weights. For Trial 2, 2011, only few significant QTLs were found for shoot traits (Table 6). Significant QTLs associated with plant fresh weight were detected in Trial 1, 2010, and Trial 2, 2010, for both trials located on chromosome 5 (at 56.0 and 92.3 cM, respectively) and on chromosome 7 at 43.6 cM (Trial 2, 2010) (Table 6). Significant QTLs associated with plant dry weight were found on chromosome 3 for Trial 1, 2011, and Trial 2, 2011, at 69 cM approx., as well as on chromosome 4 at 43 cM (Trial 2, 2010), chromosome 5 at 56.0 cM (Trial 1, 2010), chromosome 6 at 61.9 cM (Trial 2, 2011), chromosome 8 at 92 cM (Trial 1, 2010) and 68.1 cM (Trial 1, 2011) and on chromosome 9 at 52.0 cM (Trial 1, 2010) (Table 6). A significant QTL associated with NUE was found only in one trial and one chromosome (chromosome 7 at 67.0 cM in Trial 1, 2011) (Table 6).

**Table 6:** **Significant marker-trait associations (–Log_10_(P) > 3.5) for the shoot traits fresh weight (FW; g per head), dry weight (DW; g per head), and nitrogen use efficiency (NUE; g dry matter per g nitrogen taken up) at final harvest, and their position (cM) on the lettuce chromosome (Chr.) identified in each environment (Year × Trial combination) with –Log_10_(P) score, allele frequency (Allele fq.), allele effects and absolute value of the standard error (SE)**.

**Year**	**Trial**	**Trait**	**Chr**.	Marker^*^	**cM**	**−Log_10_ (P)**	**Allele fq. (%)**	Allele effect^**^	**SE**
2011	2	FW	2	LSM00500	60.7	7.25	25.0	42.5	7.82
2011	2	DW	2	LSM00500	60.7	6.82	25.0	1.69	0.32
2011	1	DW	2	LSM01045	67.6	3.78	28.6	0.66	0.17
2011	1	DW	3	LSM01342	69.4	4.99	38.0	−0.74	0.17
2011	2	DW	3	LSM01342	69.4	3.69	38.0	−1.09	0.29
2010	2	DW	4	LSM00604	43.0	4.48	34.3	2.44	0.59
2011	1	DW	4	LSM01595	72.7	3.85	17.1	−0.96	0.25
2010	1	DW	5	LSM00513	56.0	3.67	7.1	2.18	0.59
2010	1	FW	5	LSM01378	56.1	3.78	7.7	27.1	7.18
2010	2	FW	5	LSM00648	92.5	4.27	8.5	−51.5	12.75
2011	1	DW	6	LSM00165	61.9	3.83	17.5	0.77	0.20
2010	2	FW	7	LSM00610	43.6	11.59	7.8	−10.7	15.31
2011	1	NUE	7	LSM00730	67.0	4.97	23.4	1.64	0.37
2011	1	DW	7	LSM00928	68.1	4.10	31.2	0.66	0.17
2010	1	DW	8	LSM01651	92.0	5.81	8.9	2.53	0.53
2010	2	DW	9	LSM00519	52.0	3.72	10.6	−1.71	0.46

* SNPs with their flanking sequences in Supplementary Material Table S3.

** The minus sign indicates that the allele negatively affects the trait compared with the population mean.

#### Comparisons across trials and across genome

##### QTLs across trials

The QTLs detected for the below-ground traits showed reasonable consistency across trials: for instance on chromosome 7, the region around 43.6 cM was significantly associated with [NO_3_] in a 0.10 m soil layer at final harvest in Trial 2, 2010 (0.0–0.1 m; 0.1–0.2 m; 0.3–0.4 m), and in Trial 2, 2011 (0.1–0.2 m). On chromosome 8, the region around 100 cM was significantly associated with NO_3_ content over the whole soil profile in Trial 2, 2010 (intermediate sampling), and Trial 1, 2011 (final sampling).

Contrastingly, the QTLs detected for the shoot traits did not show consistency across trials for these traits: if a QTL was detected for one shoot trait in one trial, it was not found for the same trait in another trial—with the exception of a region around 68 cM on chromosome 3, which was significantly associated with dry weight at final harvest in Trials 1 and 2, 2011.

##### QTLs for multiple traits

The same QTLs were often detected for multiple traits across trials. For instance, the region around 50 cM on chromosome 5 was associated with fresh and dry weights in Trial 1, 2010. On this same chromosome, the region around 90 cM was significantly associated with fresh weight and NO_3_ left over the whole soil profile in Trial 2, 2010 (final harvest), and with water left over the whole soil profile in Trial 2, 2011 (final harvest). The region around 45 cM on chromosome 4 was significantly associated with dry weight, and with the [NO_3_] in layers 0.1–0.2 and 0.3–0.4 in Trial 2, 2010 (final harvest). On chromosome 7, the region around 43.6 cM was very significantly associated with shoot fresh weight in Trial 2, 2010, and NO_3_ left over the soil profile in the same trial. On chromosome 9, the region between 50 and 60 cM was significantly associated with the dry weight at final harvest (marker at 52 cM) and diverse below-ground traits in Trial 2, 2011, at final harvest ([NO_3_] in layer 0.2–0.3 m and 0.3–0.4 m, overall water left over the whole soil profile, and moisture content in 0.1–0.2 m and 0.2–0.3 m layers of the soil profile): the same marker was associated with several traits.

## Discussion

### Evaluation of the soil nitrate measurements method

The nitrate measurements in each soil layer were made following the method described previously by Sibley et al. (2009) and used by Kerbiriou et al. (2013b) in pot experiments. Using an ion-selective electrode enables quick and reliable nitrate measurements in the soil solution and allows the analysis of an important number of samples within a reasonable period of time and at low cost. Most studies dealing with nitrate capture at the root level use ^15^N labeling (e.g., Robinson, 2001; Popay and Crush, 2010; Yang et al., 2013, 2014), quantify root N (e.g., Ehdaie et al., 2010) or use molecular tools to quantify ${\text{NO}}_{3}^{-}$ concentrations in roots (e.g., Sorgonà et al., 2011; Wang and Shen, 2012). However, these methods can become expensive and time consuming when the objective is to quantify nitrate capture over a population of individuals. Although the range of values obtained with the electrode was sometimes large (cf. Kerbiriou et al., 2014), the values found within a sampling date were consistent across trials. The potential of this method for nitrate uptake quantification seems promising as a relatively high throughput technique for breeding programmes targeting improved resource capture below-ground.

### Timing matters

This study demonstrated that genetic control over resource capture below-ground exists, but is difficult to comprehend at early growth stages. Heritability values found at intermediate sampling for the below-ground traits were very low, if not null (Table 3) and therefore QTLs were not detected for these traits at early sampling date. Genetic variation in below-ground measurements may have been so low at early sampling because transplanted seedlings were used in this study, as opposed to direct sowing used in other studies (e.g., Johnson et al., 2000). Using transplants (a common cultivation practice in European lettuce production systems) damages the root system at transplanting and therefore may affect resource capture at early stages (Biddington and Dearman, 1985). Potentially, impaired resource capture during transplant establishment in the field may have created a residual variance due to soil conditions larger than the genotypic variance, consequently considerably lowering heritability values. On the other hand, while this was not detected in this study, Kerbiriou et al. (2013a) found that genetic variation exists in the way lettuce recovers from transplanting stress; such genetic variation was observed in resource capture and shoot traits observed at final harvest.

### Relevance of the QTLs detected

In the trials performed in this study, the variance in the dataset generated by the field conditions was so high in some cases (e.g., for the traits related to water capture) that barely any genetic variance and consequently no QTLs were detected for these traits. Although heritability values were higher for the shoot traits, the significant QTLs detected for these traits were relatively less consistent and less numerous than the significant marker-trait associations detected for the below-ground traits. One reason for this discrepancy might be that, as shown in Kerbiriou et al. (2014), the ranges of measurements obtained for the shoot traits were high, with for instance values ranging from 18.3 to 51.2 g dry matter per plant in dry conditions (Trial 1, 2010) or from 10.7 to 42.6 g dry matter per plant in wet conditions (Trial 2, 2011). The fact that the significant marker-trait associations were less consistent than expected for the shoot traits may be an artifact of the high level of G × E interactions in these trials, as was also experienced by Hartman et al. (2014) who found numerous non-overlapping QTLs among experiments correlating with stress components. Furthermore, not only the level of G × E interactions was very high, but also the physiological mechanisms regulating shoot and root growth seem to have been largely impacted by the field conditions, i.e., mechanisms regulating resource capture and use efficiency seemed specific to each field condition, making the results very difficult to generalize and extend to overall interpretations. This can be illustrated by correlation analyses carried out between shoot and root traits based on phenotypic measurements (results not shown). For instance, heavy rainfall affected Trial 2, 2011, toward the end of the experiment—just before final harvest (cf. Figure 2). This caused the nitrate in the top layers of the soil profile (0.0–0.2 m) to leach toward the lower layers of the soil profile (0.2–0.4 m); the [NO_3_] in the lower layers of the soil profile was thus larger than the [NO_3_] in the upper layers of the soil profile. This phenomenon might have impacted resource foraging for the plants, as in this trial, the shoot dry- and fresh weights were highly and significantly negatively correlated with the [NO_3_] in the lower layers of the soil profile (0.2–0.4 m). As shown by Hodge (2004), Gallardo et al. (1996a) and Kerbiriou et al. (2013b) localized root elongation happens in N-rich zones – in contrast to neighboring N-poor zones. One can thus hypothesize that during this trial, efficient N-foraging in these layers significantly contributed to shoot field performance. Such active N-foraging may have been genetically controlled as numerous QTLs were expressed on chromosome 9 around 52 cM for the below-ground traits ([NO_3_] and [H_2_O] in the lower layers of the soil profile) in this very specific environment (cf. Table 5). As shown in Figure 6, the group bearing a different allele for this marker than the rest of the population seems not to have been able to capture as much nitrate in the lower layers of the soil profile, which significantly impacted shoot growth.

In contrast, the mechanisms regulating shoot growth in Trial 2, 2010, seem different, as the correlation analysis shows that dry weight (and fresh weight to a lesser extent) was significantly negatively correlated with the [NO_3_] concentration in the different layers of the soil profile. One can imagine that in relatively warm and optimal conditions with regular rainfall, which replenished the soil profile at regular intervals (cf. Figure 2), the ability of the genotypes to display good field performance may mainly have been linked to their ability to extract nitrate from the soil profile—assuming that genotypes with a larger root system (not investigated in this study) allowing them to capture a larger amount of nitrate, would perform better than cultivars with a smaller root system. This mechanism may have been genetically controlled as, interestingly, neighboring regions on chromosome 4 were significantly associated with shoot and below-ground traits: shoot dry weight and [NO_3_] in the 0.3–0.4 layer of the soil profile were significantly associated with a marker around 40 cM on the one hand, and markers in a region between 70 and 80 cM were associated with [NO_3_] in the 0.2–0.3 m layer of the soil on the other hand (Table 5).

Overall, several regions showed to be significantly associated with below-ground traits, e.g., the region around 80 cM on chromosome 4, the region around 90 cM on chromosome 7, and the region between 50 and 60 cM on chromosome 9. The exact same regions were not identified before, although there seems to be some overlap with some regions previously identified by Uwimana et al. (2012). For instance, in our study the region between 50 and 60 cM on chromosome 9 was significantly associated with [NO_3_] of the lower layers of the soil profile in Trial 2, 2011; in Uwimana et al. (2012) a neighboring region on this chromosome was significantly associated with relative moisture content of the soil. However, the lower heritability of the data for the below-ground traits, soil moisture for instance, prevented finding QTLs for these traits. In this study, the data for soil moisture content were not corrected for water movement caused by rainfall across the soil profile; also, the data for nitrate content in the soil layers did not take into account soil moisture content data. Fitting the experimental data into a model accounting for these movements (such as “bucket-filling” models; Guswa et al., 2002) would improve the fit of the data and may therefore allow better correlation with genotypic data.

### Recommendations for future research

Given the observations above, the genotypic data used in this study may be further transformed to gain a more accurate understanding of the G × E, and exploited more in-depth to get a better insight into the mechanisms explaining the results. As this study was based on the assumption that root characteristics are strongly correlated with resource capture, it would be interesting to assess the root system architecture of the cultivars used in this association panel. It would also be interesting to investigate further how the regions identified in this study relate to each other, and how they interact with the environment, by for instance designing experiments where different stresses are applied (such as in Kerbiriou et al., 2013b). It is possible that regions located on different chromosomes are simultaneously or differentially expressed in contrasting environments.

Although these traits can be more easily measured in greenhouse experiments, such greenhouse experiments may not always reflect the reality of the field conditions. This was illustrated by a study by Hartman et al. (2012) who found different QTL patterns for fitness-related traits in lettuce (measured on shoots) in trials carried out in the field compared to greenhouse conditions.

### Implication for lettuce breeding

Most of the recent literature investigating the potential of marker use in lettuce breeding has been focusing on cultivated × wild lettuce crosses (Johnson et al., 2000; Jeuken et al., 2008; Kuang et al., 2008; Uwimana et al., 2012; Hartman et al., 2013a,b, 2014), on intra-specific crosses (Waycott et al., 1999) or recombinant inbred lines (Hayashi et al., 2012). Cultivated and wild lettuce are very different species morphologically, not only for shoot traits, but also for root traits (Uwimana et al., 2012); for instance wild lettuce develops a strong tap root which allows it to forage resources in deep soil layers, while transplanted cultivated lettuce cultivars have a small root system mostly located in the top soil layers (0.0–0.3 m; Johnson et al., 2000). Although introgressing genes from wild species into cultivated species seems a promising approach, particularly for root traits in lettuce, this is a long term strategy which requires a better understanding of the interaction patterns existing between genes located on different chromosomes. Bi-parental QTL mapping studies also tend to produce longer linkage blocks, where association panels allow a more precise localization of the regions of interest as it is based on the recombination events which occurred during the breeding history (Long et al., 2013). In this view the information provided by this study may be used immediately for breeding purposes. Indeed breeders could design new trials including diverse soil treatments (localized drought or nitrate limitation) in order to investigate if they could retrieve the QTLs found in this study and how they are expressed in controlled conditions.

However, the high frequency of the alleles shown in Tables 4, 5 for instance suggests that the genetic basis of the population chosen for the association panel may have been rather narrow. Lettuce has been bred intensively since the industrialization of the horticultural sector in the 1970s which may have reduced the genetic diversity in the commercial varieties currently available. Although population structure is visible between types (e.g., stem lettuce compared to leaf types), genetic variation within types—such as butterhead in this study—may be rather narrow. In this study, the two most different genotypes still shared about 55% of the alleles, which is a relatively high proportion. Molecular tools may therefore be useful to re-introduce genetic diversity in lettuce without the lengthy efforts of classical breeding techniques.

Furthermore, the development of more affordable and faster molecular techniques will soon allow systematic genotyping as a molecular-assisted breeding tool and might replace current techniques using genotypic markers. Indeed, sequencing the whole genome allows a more precise localization of genomic regions of interest and thus the identification of potential candidate genes regulating the expression of the trait of interest. In contrast, marker technologies can only point out potential regions of interest but do not bring much information in regards to the expression of the trait. For breeding for complex traits though, the bottleneck remains in the phenotyping. As pointed out by Johnson et al. (2000) below-ground traits are extremely difficult to evaluate and more efforts are needed to understand and quantify resource capture and use efficiency before meaningful molecular tools can be developed to breed for these traits.

## Authors contributions

PK, MK, EL, and PS conceived and designed the study. MK carried out the pre-screening of the varieties. PK carried out the field experiments and managed the data collection and data analysis. EL and PS supervised experimentation. DF and IR carried out the genotyping. CM supervised data analysis. PK, CM, TS, EL, and PS interpreted the results and wrote the manuscript.

### Conflict of interest statement

The authors declare that the research was conducted in the absence of any commercial or financial relationships that could be construed as a potential conflict of interest
